# Differential Association Between Default Mode Network Connectivity and Attachment Styles in Healthy Individuals and Crohn's Disease Patients

**DOI:** 10.1002/brb3.70620

**Published:** 2025-06-17

**Authors:** Alessandro Agostini, Sara Ventura, Silvia Tempia Valenta, Fernando Rizzello, Paolo Gionchetti, Francesca Benuzzi, Nicola Filippini

**Affiliations:** ^1^ Department of Clinical and Surgical Sciences, IRCCS S. Orsola‐Malpighi Hospital University of Bologna Bologna Italy; ^2^ Department of Biomedical and Neuromotor Sciences University of Bologna Bologna Italy; ^3^ Department of Biomedical, Metabolic and Neural Sciences University of Modena and Reggio Emilia Modena Italy; ^4^ IRCCS San Camillo Hospital Venice Italy

**Keywords:** attachment theory, Crohn's disease, default mode network

## Abstract

**Aim:**

Crohn's disease (CD) is associated with psychological disorders and insecure attachment styles, potentially reflecting the long‐lasting disease effect. Although functional magnetic resonance imaging (fMRI) studies revealed differences in CD patients relative to HC, the brain properties underlying the attachment dimensions in CD remain scarcely investigated. We carried out an fMRI study to investigate the neural substrate of the attachment dimensions in CD patients and healthy controls (HCs).

**Materials and Method:**

Nineteen CD patients and 18 HC were included in this study. All participants filled out the Attachment Style Questionnaire (ASQ) widely used to evaluate the dimensions of the attachment style and underwent an MRI protocol including structural and functional scans.

**Results:**

The ASQ scores were similar between groups. Concerning the resting fMRI data, we identified two opposite trajectories for the association between two ASQ subscales reflecting attachment insecurity and the default mode network (DMN) connectivity between the two study groups. For the HC, higher scores at the ASQ were associated with reduced DMN connectivity, whereas in CD patients were related to increased DMN connectivity. The significant clusters were located in the superior frontal gyrus, posterior cingulate, and orbito‐frontal regions.

**Conclusion:**

DMN is involved in higher mental functions including self‐consciousness and affective processes. In CD patients, the DMN modifications associated with attachment insecurity might reflect dysfunctional monitoring of the self and the significant relationships potentially involved in the development of psychological stress and decreased mentalization. Our study strengthens the notion that the attachment dimensions should be considered in the treatment of IBD and encourages novel psychotherapeutic approaches based on mentalization.

## Graphical abstract

The neural substrate of the attachment dimensions in Crohn's disease (CD) patients and healthy controls (HCs) was investigated.

In CD patients, default mode network (DMN) modifications were associated with attachment insecurity.

The DMN is involved in self‐consciousness and affective processes. DMN modifications and attachment insecurity might promote psychological disorders in CD.

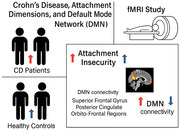

## 1 Introduction

Crohn's disease (CD) is an inflammatory, chronic bowel disease that, together with ulcerative colitis, is referred to as inflammatory bowel disease (IBD) (Lamb et al. 2019). The disease onset is around the third decade of life, and its clinical course is characterized by alternating phases of remission and inflammatory activity with debilitating symptoms such as abdominal pain, bloody diarrhea, and fever. CD causes significant psychological burden in affected patients, substantially impacting their quality of life (Lix et al. 2008; Agostini et al. 2014; Matos et al. 2021) and potentially causing symptoms such as depression or anxiety (Mikocka‐Walus et al. 2016), reduced interpersonal relationship (Collins 2020; Agostini et al. 2019), and eventually social isolation (Colonnello and Agostini 2020; Orr et al. 2024).

The quality of interpersonal relationships in patients with CD has been recently investigated in light of the attachment theory (Agostini et al. 2014; 2019; Caplan et al. 2014). This theory postulates that the infant, through interaction with caregivers, internalizes cognitive and social patterns of behavior, called working internal models (WIMs) (Bowlby 1978). WIMs guide affect and behavior in the context of interpersonal relationships throughout the life span, determining the subject's so‐called attachment style (Bowlby 1978). The attachment style has been classified as secure and insecure, with the latter further subdivided into anxious, mainly characterized by fear of abandonment and avoidant, defined by discomfort with closeness and intimacy (Bowlby 1978; Weinfield et al. 2000). Although the attachment style is thought to be quite a stable personality trait, the onset of a chronic illness and its course are believed to be adverse life events that can result in a shift toward insecurity in the patients' attachment style (Fraley et al. 2021; Waters et al. 2000; Hamilton 2000). Although longitudinal studies necessary to support this hypothesis are currently lacking, cross‐sectional studies have shown higher levels of attachment insecurity, particularly anxious and avoidant styles, relative to healthy controls (HCs) (Agostini et al. 2014; 2019; Caplan et al. 2014). These studies have associated insecure attachment in CD patients with difficulties in emotional regulation, increased perceived disease burden (Colonnello and Agostini 2020) and decreased health‐related quality of life, and heightened sensitivity to disease‐related stress (Caplan et al. 2014). Recent studies have shown reduced mentalization abilities (i.e., the capacity to understand and interpret one's own and others’ mental states such as thoughts, feelings, desires, and intentions) in CD that may contribute to emotional dysregulation and interpersonal difficulties (Agostini et al. 2019). The IBD‐derived stress (Mikocka‐Walus et al. 2019) is believed to promote the development of reduced interpersonal relationships, as shown by high levels of attachment insecurity and mentalization deficits (Colonnello and Agostini 2020). Indeed, CD represents a threatening experience that affects the attachment system of patients (Luyten et al. 2013) who in turn show behaviors that can vary from withdrawing from relationships and relying only on their own strength (in the case of a shift toward avoidant attachment) to constantly asking for closeness and help (in the case of a shift toward anxious attachment). Although attachment styles are primarily studied in the context of mental health conditions such as anxiety and depression (Jinyao et al. 2012; Dagan et al. 2018), their investigation in CD patients remains relevant even in the absence of diagnosed psychiatric disorders. Insecure attachment may negatively impact psychological resilience, thereby increasing vulnerability to psychopathology (Bifulco et al. 2002). Additionally, it may contribute to reduced social support and lower adherence to medical treatment, potentially leading to poorer disease management and progression. Attachment and mentalization‐induced modifications may lead to an increased risk of psychological disorders, decreased social support, and reduced medical compliance, potentially causing severe worsening in the disease management and progression (Colonnello and Agostini 2020; Luyten et al. 2013).

Evidence in the literature has shown that the attachment styles are related to the person's structural and functional neuronal substrate (Perlini et al. 2019). This is intriguing, particularly considering that functional magnetic resonance imaging (fMRI) studies have consistently reported brain differences in CD patients relative to HCs in brain regions related to emotion regulation, visceral sensitivity, and self‐referential processing (Agostini et al. 2013; 2017; Li et al. 2021; Yeung 2021; Liu et al. 2018) and in their patterns of functional connectivity (Li et al. 2021; Nair et al. 2019; Hopkins et al. 2021; Kornelsen et al. 2020, Zhang et al. 2022; Thapaliya et al. 2023). Task‐based fMRI studies have shown hyperactivation in the midcingulate cortex, potentially reflecting a heightened stress response in CD during cognitively challenging tasks (Agostini et al. 2017). Moreover, increased functional connectivity between the anterior cingulate cortex and sensorimotor areas, as well as between the superior frontal cortex and temporal regions, has been reported, which suggests impaired integration processing of emotional and bodily information among CD patients (Li et al. 2021). However, the brain properties underlying the attachment dimensions in CD patients remain scarcely investigated. Advanced imaging techniques have been widely used to identify brain‐related patterns associated with the different attachment styles in healthy subjects or diseased patients. Indeed, researchers have explored the specific brain features related to social interactions or emotional processing in participants with either a secure or insecure attachment style (Vrtička and Vuilleumier 2012; Nolte et al. 2013; Vrtička et al. 2012; Gobbini and Haxby 2006; M. Deng et al. 2021; Rigon et al. 2016; Vrtička et al. 2012; Vrticka et al. 2008). A specific network, called the default mode network (DMN), has been targeted as the major focus of interest. The DMN is a large, distributed neural network, encompassing several brain regions, such as the anterior and posterior cingulate, and lateral parietal lobes, and extending into mesial temporal lobe areas (Raichle et al. 2001). The DMN has been shown to be involved in higher mental functions, including self‐consciousness, affective, and introspective processes (Raichle et al. 2001; Aarts et al. 2014), which play a critical role in relation to the attachment theory (M. Deng et al. 2021). Modifications in the DMN functionality have been shown to be associated with social interactions (Long et al. 2020; Xie et al. 2016), insecure attachment (M. Deng et al. 2021; Vrtička et al. 2012; Long et al. 2020), but also with the increased risk of developing affective disorders (Aarts et al. 2014; Broyd et al. 2009). Moreover, chronic inflammation, a hallmark of CD, is increasingly recognized as influencing brain function, particularly in networks implicated in emotion regulation and self‐referential thinking. Indeed, systemic inflammation has been related to DMN functional connectivity modifications both during resting states and mentalization‐based tasks (King et al. 2022; 2021).

Here, we have carried out an MRI study, using both structural and functional measures, to investigate the DMN changes associated with the scores at the Attachment Style Questionnaire (ASQ), used to evaluate the different dimensions of the attachment style, in a group of CD patients and a group of HCs. This study may contribute to increase our understanding of the brain changes occurring in CD patients, unraveling the neural substrates underpinning the development of emotional and social disorders in CD patients.

## 2 Materials and Methods

### 2.1 Participants Recruitment

The study was approved by the local ethics committee, and all participants provided written informed consent. Fully trained physicians attending the IBD Unit of the S. Orsola‐Malpighi University Hospital in Bologna (the national referral center for research and care of patients with IBD) examined the participants and enrolled CD patients. Hematological and endoscopic data were obtained from CD patients from routine blood tests and ileo‐colonoscopies (no more than 1 week before the MRI scan). The simple endoscopic score for CD (SES‐CD) was used to evaluate the endoscopic activity. Clinicians of the IBD Unit (F.R. and P.G.) compiled the CD activity index (CDAI) (Best et al. 1976) and the SES‐CD (Lamb et al. 2019), and evaluated blood tests to characterize disease activity as follow: (A) remission: CRP < 5 g/dL, CDAI < 150, SES‐CD < 3; (B) mild activity: CRP > 5 g/dL, CDAI between 150 and 250, or SES‐CD between 3 and 6; and (C) moderate activity: CRP > 5 g/dL, CDAI > 250, or SES‐CD between 7 and 15. In addition, participants underwent a full psychological investigation (by A.A.) and were screened for neurological, psychiatric, and psychological disorders using the Mini International Neuropsychiatric Interview (MINI) (Sheehan et al. 1998), a structured interview for the *Diagnostic and Statistical Manual (DSM) of Mental Disorders* (fourth edition).

Inclusion criteria were a range of age between 18 and 55, a CD diagnosis performed at least 3 years prior to the enrollment, and being right‐handed (assessed using the Edinburgh Handedness Inventory scale [Oldfield 1971]). Exclusion criteria were current or prior history of neurological or psychological/psychiatric disease: depression, dysthymia, suicidal ideation, (hypo)manic episode, agoraphobia, social phobia, obsessive compulsive disorder, post‐traumatic stress disorder, substance and alcohol dependence/abuse, psychotic disorders, anorexia nervosa, bulimia nervosa, generalized anxiety disorder, antisocial personality disorder, having a first and/or second‐degree relative with a history of diagnosed psychiatric disorders, psychotropic medications in the previous 12 months, corticosteroids in the previous 6 months, pregnancy, clinical indications for forthcoming surgery, claustrophobia, and presence of metallic implants in the body. Age, gender, and educational level were collected during the recruitment stage. Moreover, according to the Montreal classification (Satsangi et al. 2006), information related to the age at diagnosis, disease location, and disease behavior, and furthermore perianal disease, extraintestinal manifestations, previous history of intestinal surgery, treatment with biologics, and possible maintenance treatments, were also recorded. A total of 19 CD patients were included in this study.

A group of 18 right‐handed HCs was also recruited, among the staff of the University of Bologna, as a control group. They underwent the same screening procedures as the CD patients and the same inclusion and exclusion criteria, except for the CD‐specific exams. An additional exclusion criterion was a first‐degree relative with CD.

### 2.2 Psychometric Analysis

All participants filled out the ASQ (Feeney 2000). The ASQ is a self‐report questionnaire containing 40 items designed to assess attachment dimensions. ASQ is a standard, validated, and widely used measure of attachment in adults. Each of the 40 items corresponds to a statement (i.e., “I prefer to depend on myself rather than other people”), and participants must rate if they agree or disagree with it by using a six‐point Likert scale in which 1 corresponds to “*totally disagree*” and 6 to “*totally agree*.” The ASQ contains five subscales: (ASQ1) confidence, describing secure attachment; (ASQ2) discomfort with closeness and (ASQ3) relationships as secondary, both assessing attachment avoidance; and (ASQ4) need for approval and (ASQ5) preoccupation with relationships, both assessing attachment anxiety. High values in the ASQ1 scale reflect high attachment security, while high values in the other four subscales reflect high attachment insecurity.

### 2.3 MRI Data Acquisition and Preprocessing

#### 2.3.1 MRI Data Acquisition

Scanning was performed at the Ospedale Civile di Baggiovara, Modena, using a Philips Intera system at 3.0 Tesla equipped with an eight‐channel head‐coil. The neuroimaging protocol included functional and structural sequences.

*Structural MRI*: 3D high‐resolution T1‐weighted MR images were acquired using an MPRAGE sequence (TR = 9.9 ms, TE = 4.6 ms, 170 sagittal slices, voxel dimension = 1 mm isotropic).

*Resting‐state fMRI (rs‐fMRI)*: Whole‐brain functional imaging was performed using a gradient echo EPI sequence (TR = 2000 ms, TE = 35 ms, field of view = 240 mm, voxel dimension = 3 mm × 3 mm × 4 mm, number of volumes = 240). For the resting‐state scan, subjects were instructed to lie in dimmed light with their eyes open, think of nothing in particular, and not to fall asleep.

#### 2.3.2 MRI Data Processing

Data analysis was carried out using FSL tools (FMRIB Software Library, www.fmrib.ox.ac.uk/fsl).

*Structural MRI*: Preprocessing for structural images included the following steps: (A) reorientating images to the standard (MNI) template, (B) bias field correction, (C) brain extraction, and (D) brain tissues segmentation using FMRIB's Automated Segmentation Tool (FAST) that allows generating maps and deriving measures of total GM, white matter (WM), and cerebrospinal fluid (CSF) for each individual subject. Whole brain analysis was carried out using a voxel‐based morphometry‐style analysis (FSL‐VBM) (Douaud et al. 2007), using default settings. In brief, brain extraction and tissue‐type segmentation were performed, and resulting GM partial volume images were aligned to the MNI standard space using first linear (FLIRT) and then nonlinear (FNIRT) registration tools. A study‐specific GM template was created. Images were averaged, modulated, and smoothed with an isotropic Gaussian of 6 mm full width at half max (FWHM), and the GM images were reregistered to the study‐specific GM template, including modulation by the warp field Jacobian.

*rs‐fMRI*: fMRI analysis of resting state data was carried out using fMRI Expert Analysis Tool (FEAT) v. 6.00 (Woolrich et al. 2004). Individual pre‐statistical processing consisted of motion correction, brain extraction, and spatial smoothing using a Gaussian of FWHM (full width at half maximum) 5 mm, and high‐pass temporal filtering with a cutoff of 100 s (0.01 Hz). fMRI volumes were registered to the individual's structural scan and standard space images using both FLIRT and FNIRT registration tools. As the signal derived from rs‐fMRI is affected by the presence of artifacts, many of which have a spatial and/or spectral overlap with resting state networks (RSNs) of interest, here, we have used a tool called FIX (Griffanti et al. 2015) to denoise functional images from the spurious signal and increase the possibility of identifying markers of effective connectivity. Preprocessed and denoised functional data containing 240 time points for each subject were temporally concatenated across subjects in order to create a single 4D dataset. The number of components was fixed at 25 based on an initial analysis of the population using model order estimation. The subject‐dependent effect sizes identified in the initial analysis suggested that only 25 components were significantly nonzero on average. RSNs of interest covered the entire brain and were selected using spatial correlation against a set of previously defined maps. The between‐subject analysis of the resting data was carried out using a regression technique (dual regression), which allows for voxel‐wise comparisons of resting functional connectivity maps (Filippini et al. 2009).

### 2.4 Psychometric and MRI Data Statistical Analysis

Group comparison of continuous variables (sociodemographic and ASQ‐derived values) was performed using *t*‐tests, whereas the chi‐square (*χ^2^*) test was used for a binary variable (gender). Statistical analysis was performed using SPSS software (v 25.0).

With regard to the imaging data, the five values derived from the ASQ were included as separate covariates of interest for the general linear model (GLM) analyses on MRI data. The objective was to identify those brain regions reflecting group differences (i.e., differences in “correlation slopes”) associated with the ASQ‐derived scores.

Voxel‐wise GLM was applied on structural and RSN maps using randomize, a permutation‐based nonparametric testing (5000 permutations) (Nichols and Holmes 2002), and threshold‐free‐cluster‐enhancement (TFCE) for clusters identification (Smith and Nichols 2009). An FWE‐corrected cluster significance threshold of *p* < 0.05 was applied to the suprathreshold clusters.

## 3 Results

### 3.1 Participants

Note that *t*‐tests and chi‐square analyses revealed that HC and CD patients did not significantly differ in terms of years of age, education, and gender (M/F) (Table 1). According to the Montreal classification, four CD patients were categorized as A1, and 15 were categorized as A2. Regarding disease location, eight patients had ileal involvement (L1), and 11 had ileo‐colonic involvement (L3). At the time of recruitment, disease behavior was classified as non‐stricturing and non‐penetrating (B1) in 10 patients, B1p in two, stricturing (B2) in three, penetrating (B3) in one, and B3p in three patients. Clinically, 14 patients were in remission with a CDAI < 150, four had mild disease activity with a CDAI from 150 to 250, and one had moderate activity (CDAI > 250). Using the SES‐CD scores, 14 patients were classified in remission (SES‐CD < 3), four had mild endoscopic activity (SES‐CD, 3–6), and one had moderate activity (SES‐CD, 7–15). Seven patients experienced extraintestinal manifestations, including six with arthralgia and one with erythema nodosum. Five patients had undergone IBD‐related surgeries, with two requiring ostomy. Current treatments included biologics (infliximab and adalimumab) for seven patients, 5‐aminosalicylic acid for seven, and azathioprine for five.

**TABLE 1:** Sociodemographic and psychometric measures of the two study groups.

	Controls, *N* = 18	CD patients, *N* = 19	*p*‐value
Sociodemographics			
Age (Y)	30.74 (±6.06)	28.33 (±5.47)	0.21
Education (Y)	15.53 (±2.72)	16.06 (±2.51)	0.54
Sex (M/F)	8/10	7/12	1
Disease duration (Y)	NA	10.58 (±5.63)	
ASQ			
ASQ1	33.61 (±4.38)	33.37 (±4.07)	0.86
ASQ2	32.89 (±7.34)	29.05(±7.78)	0.13
ASQ3	13.72 (±4.19)	12.52 (±4.10)	0.39
ASQ4	19.33 (±5.88)	18.15 (±5.44)	0.53
ASQ5	25.55 (±5.40)	25.16 (±5.33)	0.82

*Note*: In columns, values are expressed as mean (± standard deviation). Numbers of subjects are expressed as *N*. *t*‐tests were used for continuous variables comparisons, whereas the chi‐square test was used for the gender variable.

Abbreviations: ASQ, Attachment Style Questionnaire; ASQ1, confidence; ASQ2, discomfort with closeness; ASQ3, relationships as secondary; ASQ4, need for approval; ASQ5, preoccupation with relationships; M/F, male/female; NA, not applicable; Y, years.

### 3.2 Psychometric Data Analysis

There were no significant differences between patients with CD and HCs in the scores derived from the ASQ. Note that *t*‐tests analyses revealed no differences in the self‐report measures of attachment security: ASQ confidence (*p* = 0.86); attachment avoidance: ASQ discomfort for closeness (*p* = 0.13) and ASQ secondary with relationships (*p* = 0.39); and attachment anxiety: ASQ need for approval (*p* = 0.53) and ASQ preoccupation with relationships (*p* = 0.82) (Table 1).

### 3.3 Imaging Data Analyses

*Structural MRI*: Voxel‐wise GLM analysis shows no significant differences between the two study groups in the relationship between brain morphological measures and any of the five variables of interest.

*rs‐fMRI*: A trained neuroscientist (N.F.) visually inspected preprocessing of data from all 37 participants in order to ensure accurate registration. The optimal threshold for FIX was determined to be 30, with a median true positive rate (TPR) of 95.3% (95.8%) and a true negative rate (TNR) of 90.7% (92.5%). These values meet or exceed the recommended thresholds that are TPR > 95% and TNR > 70%.

**FIGURE 1:**
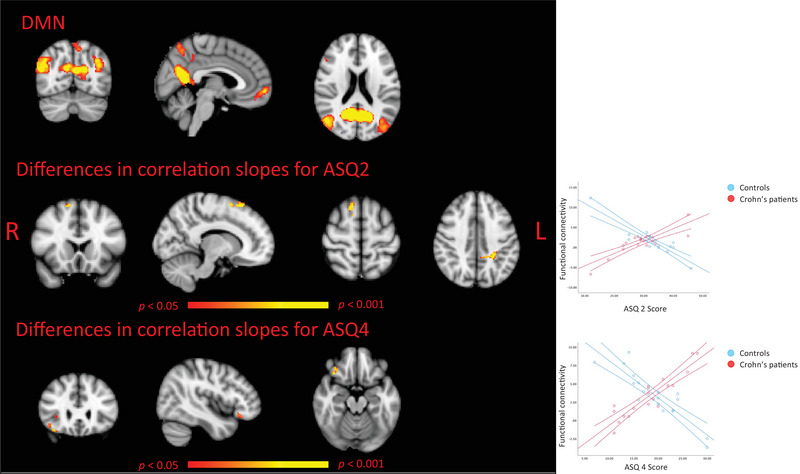
The images illustrate differences between Crohn's disease (CD) patients and healthy controls (HCs) in voxel‐wise correlations between the scores derived from the Attachment Style Questionnaire (ASQ) subscales and default mode network (DMN). The top panel shows the DMN group map. The middle panel highlights voxels where the correlation between the scores of the dimension ASQ2 (discomfort with closeness) and DMN connectivity strength differs between groups, with significant regions located in the superior frontal gyrus and the posterior cingulate extending into the lateral parietal area. The bottom panel depicts voxels with group‐related differences in the correlation between the scores obtained in the dimension ASQ4 (need for approval) and DMN connectivity, primarily in the orbito‐frontal region. For significant clusters, scatterplots are provided to illustrate the relationship between extracted brain region values and ASQ2 and ASQ4 scores, with red dots representing CD patients and blue dots representing healthy controls. All images display results at *p* < 0.05, corrected for multiple comparisons (red‐to‐yellow color scale). “R” indicates the brain's right hemisphere, while “L” indicates the left.

Out of the 25 derived RSNs, we focused our analysis on the DMN. A group‐related differential association between two of the variables of interest and functional connectivity within the DMN was found by GLM analyses. Specifically, increased DMN connectivity was observed in CD patients with higher scores in the dimension ASQ2 (discomfort for closeness) compared to HC, who exhibited the opposite trend. This effect was found in two clusters: one in the right superior frontal gyrus (64 voxels, *T*‐max: 6.12, MNI coordinates: 39‐68‐67) and another in the left precuneus extending into the left superior parietal gyrus (61 voxels, *T*‐max: 4.66, MNI coordinates: 49‐41‐56) (see Figure 1 in the center). Similarly, we observed increased DMN connectivity associated with ASQ4 (need for approval) scores in CD participants relative to the HC group, for whom the opposite pattern was observed, in a cluster largely located in the right orbital frontal cortex (63 voxels, *T*‐max: 5.14, MNI coordinates: 28‐80‐27] (see Figure 1 below).

## 4 Discussion and Conclusion

In the present study, we compared the scores at the ASQ and their association with imaging‐derived measures in a group of CD patients relative to a group of HC.

### 4.1 Psychometric and Imaging Data Analyses

Psychometric data analysis revealed no group‐related differences in the ASQ performance for any of the five subscales. This contrasts with previous findings available in the literature (Agostini et al. 2014; 2019). However, the small enrollment of participants and the exclusion of CD patients with symptoms of psychological suffering may at least in part explain these results. Similarly, no group‐related differences were observed for structural MRI data. Conversely, with regard to the resting fMRI data, we were able to identify two opposite trajectories for the association between the two ASQ subscales’ scores (ASQ2 and ASQ4, reflecting attachment insecurity) and DMN connectivity between the CD patients and the HC group. In detail, for the HC group, higher scores at the two subscales were associated with reduced DMN connectivity, whereas in CD patients, higher scores at the ASQ scales were related to increased DMN connectivity. The significant clusters were located in cognitive‐control areas, namely the superior frontal gyrus and the posterior cingulate for the ASQ2 subscale, and the orbito‐frontal region for the ASQ4 subscale. These results suggest a differential neural substrate in HC and CD patients associated with their perceived attachment style.

### 4.2 Results Interpretation

To date, imaging studies have proved to be sensitive in identifying brain functional differences in IBD patients compared to HC, particularly when facing emotional and stressful stimuli (Agostini et al. 2013; 2017), which are known to be modulated by the disease. The quality of interpersonal relationships is another aspect influenced by the chronic course of the disease, and vice versa, but no previous investigation has evaluated whether this is associated with specific neural patterns.

Here, we specifically targeted the DMN for two reasons: (A) studies have shown that it plays a crucial role in relation to the attachment theory (Vrtička and Vuilleumier 2012; M. Deng et al. 2021; Vrtička et al. 2012; Long et al. 2020), and (B) reduction in the global DMN connectivity was consistently reported in CD patients relative to HC (Yeung 2021; Kornelsen et al. 2020; J. Deng et al. 2023; Thomann et al. 2017; 2021), which suggests that the DMN may represent an ideal target network to investigate the neural correlates of changes associated with disease‐derived symptoms. Our findings in the HC group (i.e., reduced DMN connectivity associated with higher ASQ‐related scores) are supported by a recent study where healthy participants with perceived dysfunctional parenting (that is associated with insecure attachment styles) showed a reduction in the DMN connectivity relative to a group of healthy participants with perceived functional parenting (Adenzato et al. 2019). Authors interpreted these findings as reflecting a reduced ability to mentalize the relational experiences with significant others (Adenzato et al. 2019). Conversely, the increased DMN connectivity related to higher ASQ discomfort with closeness and need for approval scores, observed in the CD group, may suggest that DMN modifications associated with attachment insecurity could reflect the role played by chronic stress and inflammation. This seems to be supported by evidence suggesting that DMN alterations have been associated with chronic stress, peripheral inflammation, and early adverse experiences, which in turn could affect social cognitive processing, mentalization, and ultimately the attachment dimensions (King et al. 2022; 2021). Indeed, the constant threat of inflammatory reactivation induces patients to repetitively and often anxiously monitor the potential onset of visceral symptoms (Mikocka‐Walus et al. 2016; Thomann et al. 2017). Although preliminary, our findings may expand these previous interpretations in the context of interpersonal relationships. The increased DMN connectivity that we found associated with attachment insecurity might reflect the increased tendency of CD patients to monitor themselves and their significant relationships since the self and the self in relation to others are experienced by patients as threatened by the recurrent symptoms of the chronic disease. Indeed, our results concerned the ASQ “discomfort with closeness” and “need for approval” subscales. The former might reflect the difficulty in intimate relationships plausibly associated with the condition of being chronically ill with embarrassing symptoms such as recurrent diarrhea. The latter may show the patient's need to be accepted by significant others despite the chronic condition and disabilities caused by the disease. Another plausible explanation for the increased DMN connectivity observed in CD patients is that it may reflect altered emotion regulation processes, which have been previously identified in IBD using both neuroimaging (Yang et al. 2024) and non‐neuroimaging investigations (Mikocka‐Walus et al. 2016).

### 4.3 Study Limitations

The main limitation of our study was the limited number of participants recruited, which may have hindered the power of detecting significant differences for psychological variables and also prevented the stratification of the sample to allow for a direct comparison between securely and insecurely attached CD patients. Due to the limited number of CD patients, it was not feasible to stratify the sample based on disease severity or to explore potential relationships between inflammatory mediators and brain activity. Further research involving adequately powered samples, allowing for the stratification of patients with CD based on disease activity status (active vs. remission) and the presence or absence of psychopathological symptoms, is warranted to elucidate the complex interplay between insecure attachment, dysfunctional connectivity within the DMN, and systemic inflammation. In particular, longitudinal studies investigating the potential changes of attachment dimensions in CD, as well as fMRI investigations employing task‐based paradigms targeting emotional processing, attachment, and mentalization, are needed. Finally, here, we have used a semi‐structured interview to exclude the presence of mood disorders in our participants, but future studies should also include psychometric questionnaires measuring relevant symptoms such as depression and anxiety and potentially be suitable for also assessing subclinical levels of distress in patients with CD.

### 4.4 Conclusion

Our study, though preliminary, provides intriguing insights for future research on the neural correlates associated with attachment style in IBD patients. Based on our results, we hypothesize that the CD course might affect patients’ self‐awareness, promoting an increased monitoring of the self. Dysfunctional self‐monitoring processes are associated with potential neural vulnerability to psychological disorders that are common in IBD and are related to worsening of the disease. Indeed, psychological stress is believed to modulate inflammatory processes and affect interpersonal relationships, eventually leading to social isolation. The present study further strengthens the notion that the attachment dimensions should be considered in the treatment of IBD.

## Author Contributions

**Alessandro Agostini**: conceptualization, investigation, writing – original draft, supervision, writing – review and editing. **Sara Ventura**: data curation. **Silvia Tempia Valenta**: data curation. **Fernando Rizzello**: investigation, data curation. **Paolo Gionchetti**: investigation, data curation. **Francesca Benuzzi**: investigation, methodology, software, data curation. **Nicola Filippini**: conceptualization, methodology, writing – review and editing, formal analysis, software, supervision.

## Ethics Statement

The study was approved by the local ethics committee (Prot n 2453/CE N. di pratica CE 45/14), Comitato Etico Provinciale di Modena Azienda Ospedaliero‐Universitaria.

## Consent

All participants provided written informed consent.

## Conflicts of Interest

The authors declare no conflicts of interest.

## Peer Review

The peer review history for this article is available at: https://publons.com/publon/10.1002/brb3.70620.

## Data Availability

Data and materials are available at request.
